# Recovery of High Interference Memory in Spite of Lingering Cognitive Deficits in a Longitudinal Pilot Study of Hospitalized Depressed Patients

**DOI:** 10.3389/fpsyt.2020.00736

**Published:** 2020-07-24

**Authors:** Xue Han, Yingga Wu, Yanfeng Zhong, Suzanna Becker

**Affiliations:** ^1^School of Psychology, Northeast Normal University, Changchun, China; ^2^Tongliao Mongolian High School, Tongliao, China; ^3^Changchun Mental Hospital (Changchun Sixth Hospital), Changchun, China; ^4^Department of Psychology, Neuroscience and Behaviour, McMaster University, Hamilton, ON, Canada

**Keywords:** depression, cognitive impairments, neurogenesis, high interference memory, mnemonic similarity task, remission

## Abstract

**Background:**

Major depressive disorder has deleterious impacts on mood, cognition, and many functions of daily life. Even after remission of mood symptoms, patients frequently report persistent cognitive deficits. By contrast, the neurogenic theory of depression posits that recovery from depression is dependent upon a restoration of neurogenesis. The present study was designed to test this prediction by assessing performance in MDD in-patients on a broad battery of cognitive tasks including the Mnemonic Similarity Task, a high interference memory test that is a putative correlate of neurogenesis. We predicted that remitted patients should exhibit recovery of function on this task, even though they may show residual deficits on other cognitive tasks.

**Methods:**

18 hospitalized patients diagnosed with MDD and 22 healthy control participants matched for age, sex, and education completed a battery of mood and cognitive tests at two time points. Patients completed their baseline assessments when first admitted to hospital and repeated the same assessments upon remission, typically 4–5 weeks later and just prior to their release from hospital. Control participants were tested at baseline and 4–5 weeks later on the same assessment battery, which included the BDI-II, BAI, Cohen’s PSS, Mnemonic Similarity Task, and several sub-tests adapted from the CANTAB.

**Results:**

At baseline, MDD patients were impaired relative to controls on the MST and many other cognitive tasks. Upon remission, patients’ MST scores did not differ from those of healthy controls, although patients were still impaired on Pattern Recognition Memory, Spatial Recognition Memory, Delayed Matching to Sample and Paired Associates Learning relative to healthy control participants.

**Conclusion:**

The lingering memory deficits observed in remitted patients with MDD observed here are broadly consistent with findings in the literature. Importantly, however, remitted patients showed recovery of cognitive function on the Mnemonic Similarity Task. This is the first study that we are aware of to report recovery of function on a high interference, putatively neurogenesis-dependent memory test in a longitudinal sample of hospitalized MDD patients from admission to remission. Our findings are consistent with the neurogenic theory of depression, which posits that a restoration of neurogenesis is linked to recovery from depression.

## Introduction

According to the World Health Organization, more than 264 million people suffer from depression, and it is the leading cause of disability worldwide. Major depressive disorder (MDD), as defined by the DSM-5 (1), affects an individual’s mood, concentration, and decision making. Meta-analytic studies have revealed that depressive patients show deficits on a wide range of cognitive functions, including attention, processing speed, executive functions, cognitive flexibility, attentional switching, visual learning, and memory (2–5). Moreover, relative to healthy control participants, unipolar depressive patients showed significantly slower and poorer performance when the visuospatial attention task complexity increased (6), suggesting that whether or not cognitive impairments are observed may depend on the task difficulty. Additionally, those who suffer from MDD frequently report impairments in many aspects of daily life functioning (7–9).

Even after recovering from MDD, remitted patients frequently experience lingering cognitive deficits. A meta-analysis of 252 studies found that patients in remission from a major depressive episode exhibited small to medium sized deficits in processing speed, visual selective attention, working memory, verbal learning, and executive functions, and large deficits in long-term memory measures including recall and recognition (10). Similarly, a systematic review and meta-analysis that only included studies using the Cambridge Neuropsychological Test Automated Battery (CANTAB) found that across studies, patients with depression were impaired in executive functions, memory, and attention compared to healthy control participants (11). Furthermore, even in remitted patients, executive function and memory scores were still worse than those of controls (11). Patients in remission from depression also show lingering cognitive emotional biases; for example, they show an attentional bias toward negative words, are more likely to recall negative autobiographical memories, and more likely to recall overly generalized memories of positive events (12). The role of emotional factors in modulating cognitive processes is well documented [for example, in face processing (13, 14)] and is an important area for future research in depression.

While meta-analyses reveal lingering cognitive deficits in remitted MDD patients in the majority of studies, there is a high degree of variability in these findings. A major source of this heterogeneity is the variation in the number of previous depressive episodes; patients who have experienced more depressive episodes exhibit greater deficits upon remission (10). Consistent with the lingering memory deficits in multi-episode remitted patients, structural neuroimaging studies have revealed a negative association between hippocampal volume and total lifetime duration of illness in major depression (15, 16). Another reason for the inconsistencies in the literature on cognitive function in remitted patients may be that a wide range of cognitive tasks of varying difficulty levels were used in different studies.

In addition to the loss of hippocampal volume in MDD patients, another important neural correlate may be the loss of hippocampal neurogenesis. In the past decade, a mounting body of evidence from rodent models has linked hippocampal neurogenesis to depression (17–20). In rodents, reduced neurogenesis is both a consequence of chronic stress exposure (21) and a vulnerability that predisposes animals to develop depressive symptoms when later subjected to stress (22). On the other hand, the anti-depressant effects of pharmacological treatments (SSRIs) and exercise may be dependent upon the restoration of neurogenesis (23, 24). Thus, the neurogenic theory of depression (17) posits that reduced neurogenesis increases vulnerability to developing depression, while increased neurogenesis is required for overcoming depression. While neurogenesis may not explain all aspects of depression, and a wider range of plasticity mechanisms are likely at play (25), the evidence that neurogenesis does play a causal role in both the pathogenesis of and recovery from depression in rodent models is compelling. However, in the absence of a non-invasive measure of neurogenesis, there is not yet any direct confirmatory evidence in humans. Nonetheless, correlational evidence has identified several high interference memory tests in human studies as putatively neurogenesis-sensitive, because they are similarly impacted by factors that alter neurogenesis levels in rodents. One task in particular, the Mnemonic Similarity Test (MST) (26), is a strong contender as a putative correlate of neurogenesis in humans. The MST was formerly called the Behavioral Pattern Separation task but was re-named MST to underscore that “pattern separation” is a property of neural coding and cannot be assessed behaviorally [for a discussion, see Becker (27)]. Importantly, deficits on the MST are associated with elevated scores on scales of stress, depression and binge drinking, and with increased age (28–32), while several weeks of high intensity exercise improved MST scores (28). Gandy and colleagues proposed that the MST could be used as a possible tool to test impaired hippocampal neurogenesis in major depressive disorder (33). If the neurogenic theory of depression is correct, patients who are in a depressed state should be relatively impaired on the MST, while patients in a remitted state should exhibit improved MST scores even though they may have other lingering cognitive deficits.

The present study was designed to assess the primary question of whether recovery from depression is linked to a restoration of high interference memory performance and secondarily, whether other cognitive deficits would be apparent at baseline and recovery. Therefore, a battery of cognitive assessments was administered to patients hospitalized for depression and healthy control participants at two time points, when the patients were first admitted to hospital and upon remission, just prior to their discharge from hospital (typically 4–5 weeks later). Healthy control participants, matched for age, sex, and education, were tested at similar intervals of 4–5 weeks from baseline to end of study. We hypothesized that patients would exhibit broad cognitive deficits at baseline, and upon remission, they would show recovery of high interference memory function—a putative correlate of neurogenesis—in spite of deficits in other cognitive domains.

## Materials and Methods

### Participants

Forty people participated in our study: 18 patients were recruited *via* consecutive sampling from a psychiatric hospital (eight males and 10 females) and 22 healthy controls matched for age, sex, and education (eight males and 14 females). Demographic variables including age, sex, and years of education are summarized in **Table 1**. In-patients were in an “open ward” in which patients were freely permitted to leave their rooms but had to stay within the ward, and family were permitted to visit. There was no significant difference between the two groups in terms of age (t = 1.860, df = 38, *p* = 0.071), or education level (t = −1.911, df = 38, *p* = 0.064). The criteria for recruiting patients were as follows: All of the patients were admitted to the hospital for the first time with a diagnosis of major depressive disorder, no other diagnosed comorbidities or physical health conditions, and they were able to read and understand instructions and perform the cognitive tasks. The clinical diagnoses were made by the patients’ psychologists in the hospital. Their assessments included clinical interviews and the completion of several questionnaires. Patients received treatment as usual in the course of the study, including pharmacotherapy and/or psychotherapy. Both patients and healthy controls completed Beck Depression Inventory II as a measurement of their depressive mood in our study. No other exclusion criteria were applied to the controls. All participants voluntarily chose to join the study, provided written consent, and were free to withdraw at any time. No patients or healthy control participants dropped out of the study. The study was reviewed and approved by the ethics committees in the Changchun Mental Hospital and Northeast Normal University (2017002).

**Table 1:** Demographic information.

	Patients	Healthy Controls
Age	31.22 ± 11.42	26.36 ± 4.08
Male/Female	8/10	8/14
Years of Education	12.33 ± 2.22	13.45 ± 1.47
Clinical Diagnosis	Major Depressive Disorder	None
Medications	Fluoxetine, Sertraline, and/or Paroxetine	None
Psychotherapies	Sandbox Therapy, Cognitive Behavioral Therapy, and/or Hypnosis, but no Electroconvulsive Therapy	None

### Procedures

The Beck Depression Inventory II (BDI) and Beck Anxiety Inventory (BAI) (licensed from Psychological Corp), Cohen’s Perceived Stress Scale (PSS) (freely available at http://www.psy.cmu.edu/~scohen/scales.html), the Mnemonic Similarity Task (MST) (26), and several sub-tests adapted from the Cambridge Neuropsychological Test Automated Battery (CANTAB) and implemented in e-prime were administered to participants at the two time points, baseline and end of study. From the CANTAB battery, we used the Motor Screening Task (MOT) to screen for sensorimotor deficits or lack of comprehension of task instructions, Rapid Visual Information Processing (RVIP) to assess executive functions (working memory and sustained attention), and Pattern Recognition Memory (PRM), Paired Associates Learning (PAL), Delayed Matching to Sample (DMS) and Spatial Recognition Memory (SRM) to test long-term memory. The baseline battery was administered to each patient upon their initial admission into hospital, while their post-recovery test battery was administered when the patient’s doctor had recommended their release from hospital, just prior to their discharge, with an average interval of 4.49 ± 0.427 weeks between the two testing points. Healthy control participants were tested across a similar interval (4.55 ± 0.433 weeks), which was matched with that of the patients. Patients were not tracked by the hospital or followed up by the same doctors post-discharge, so it was not possible to conduct long-term follow-up with these patients. For all cognitive tests, participants’ recognition accuracy for the correct responses was recorded and analyzed.

#### Motor Screening Task (MOT)

In this task, a colored ‘X’ was repeatedly displayed on the screen at different locations, and the participants were asked to click on it in each of the 15 trials. This task was used to assess, in a preliminary analysis, whether there was any impairment in motor function and/or the capacity to follow task instructions.

#### Rapid Visual Information Processing (RVIP)

In the middle of the screen, a white square was displayed and within the square, digits from one to nine appeared one at a time in pseudo-random order. A target sequence of three digits was also displayed, and participants were asked to click the mouse as soon as possible when they the target sequence had appeared. A total of 10 of these trial sequences were presented. The average accuracy across 10 trials was analyzed.

#### Pattern Recognition Memory (PRM)

This task had two blocks with a study phase and a test phase in each. During the study phase, a series of patterns was displayed, one at a time, in a square in the center of the screen, and the participants were asked to remember these patterns. During the test phase, two patterns appeared simultaneously and the participants were asked to choose the one that they had seen in the study phase. In each block, participants were required to learn 12 patterns in the study phase and to respond to 12 pairs of patterns in the test phase. The average accuracy across 24 trials was analyzed.

#### Spatial Recognition Memory Task (SRM)

There were four blocks each consisting of a study phase and a test phase with five trials in each. In each study phase, five squares were displayed sequentially at different locations on the screen, and participants were asked to remember their locations. In each of the test phases, there were two squares displayed simultaneously at different locations on the screen, and the participants were asked to choose the square in the location where they had seen the squares during the study phase. The average accuracy across 20 trials was analyzed.

#### Delayed Match to Sample (DMS)

In this task a target pattern would appear, and then four test patterns, one of which matched the target, would appear after a delay that ranged from 0 to 12,000 ms, thus creating varying difficulty levels. The patterns had irregular geometric shapes and combinations of colors and were not readily encoded using verbal or categorical strategies. In level one, the target pattern and four testing patterns appeared simultaneously. In level two, immediately after the target pattern disappeared, the four testing patterns appeared. In level three, there was an 8,000-ms delay between the disappearance of the target pattern and the appearance of the four testing patterns, and in level four, there was a 12,000-ms delay. In each case, participants had to choose the testing pattern that was the same as the target pattern. If they made an incorrect choice, they had to make another choice until they found the target. The average accuracy of the first choice at the longest delay (the most difficult level) was analyzed.

#### Paired Associate Learning (PAL)

This task had five difficulty levels. For the first four levels, during the learning phase, six squares were displayed in a circular configuration around the screen. In each trial, one or more of these squares disappeared, sequentially, to reveal a pattern, and the participants were asked to remember these patterns and their corresponding locations. Level one had only one square with a pattern in it, level two had two squares with patterns, level three had three squares with patterns, and level four had six squares with patterns. Thus, in level four, participants had to remember all six patterns and their locations in one trial. During the testing phase, in each trial, there was one square with a pattern displayed in the center of the screen, and the participant had to use mouse to click the location where they had seen this pattern during the learning phase. In level five, everything was the same as in level four except there were eight squares concealing eight different patterns to be remembered. In all levels, if the participants correctly chose the locations of all patterns, the level was completed; otherwise, they had to redo this level. The average accuracy of the first choice across the last two levels (PAL-6 and PAL-8) was analyzed.

#### Mnemonic Similarity Test (MST)

Participants studied a series of images of everyday objects and were subsequently tested on their recognition memory of these images among a set of test images. The test images were of three types, repeats of previously studied items, foils that were not previously seen, and lure items which were perceptually similar but not identical to previously studied items. There were four blocks of trials, with a study phase and a test phase in each block. During each study phase, participants saw a set of objects one at a time and were asked to remember the objects presented. During each test phase, participants saw another set of objects one at a time and were asked to respond “New” to foil objects, “Old” to repeated objects, and “Similar” to lure objects. There were 120 New, 44 Old, and 44 Similar trials in total. We analyzed the Lure Discrimination Index (LDI) (34), a bias-corrected measure of lure discrimination performance which is the percentage of lures correctly identified as “Similar” minus the percentage of foils incorrectly identified as “Similar”.

### Analyses

SPSS 25 was used for all analyses. No data imputation methods were employed for missing data. To check for any impairment in motor function or capacity to follow task instructions in patients, a preliminary ANOVA of scores on the motor screening task was conducted. To assess differences in mood scores between patients and controls at the two time points, a two-way group (controls *versus* patients) by time (baseline *versus* end of study, repeated measures) MANOVA was conducted with BDI, BAI, and PSS scores as dependent variables. Similarly, to assess for differences in cognitive performance, a two-way (group by time) repeated measures MANOVA was conducted on RVIP, PRM, SRM, DMS, PAL, and MST scores. In all *post hoc* t-tests, Bonferroni correction of alpha values was used to adjust for multiple comparisons.

## Results

Preliminary analyses of MOT scores (shown in **Table 2**) were conducted to assess potential impairments in motor function or the ability to follow task instructions. Repeated measures ANOVAs revealed no significant main effects or interactions on either accuracy (time: F(1,38) = 1.229, *p* = 0.274, η^2^ = 0.031, group: F(1,38) = 2.319, *p* = 0.136, η^2^ = 0.058, time * group: F(1,38) = 1.229, *p* = 0.274, η^2^ = 0.031), or processing speed (time: F(1,38) = 0.064, *p* = 0.801, η^2^ = 0.002; group: F(1,38) = 0.490, *p* = 0.488, η^2^ = 0.013; time * group: F(1,38) = 3.709, *p* = 0.062, η^2^ = 0.089). Out of 15 MOT trials, at baseline, two patients made one error and the remaining 16 patients made zero errors, demonstrating adequate sensorimotor ability and comprehension of touchscreen procedures to perform all tests. MOT scores were excluded from subsequent analyses. Means and standard errors of mood scores and accuracy on cognitive tests are shown in **Table 3**.

**Table 2:** Means and standard deviations of accuracy, processing speed, and number of errors for motor screening task.

Motor Screening Task	18 Patients		22 Healthy Control Participants	
	Pre-Test	Post-Test	Pre-Test	Post-Test
Accuracy	99.26% (2.16%)	99.63% (1.58%)	100% (0)	100% (0)
Processing Speed	1,225.40 (417.95)	1,126.89 (278.11)	1,070.48 (244.67)	1,146.08 (382.05)
Number of Errors	0.11 (0.32)	0.056 (0.24)	0 (0)	0 (0)

### Mood Scores

On measures of mood (BDI, BAI, PSS), a repeated measures (group × time) MANOVA revealed significant main effects of groups, *F* (3, 36) = 4.532, *p* = 0.009; Wilk’s Λ = 0.726, Observed Power = 0.846, and time, *F* (3, 36) = 15.386, *p* < 0.0005; Wilk’s Λ = 0.438, Observed Power = 1, and a significant group by time interaction, *F* (3, 36) = 9.966, *p* < 0.0005; Wilk’s Λ = 0.546, Observed Power = 0.996. Pairwise Bonferroni-corrected comparisons of marginal means within the MANOVA analysis revealed a significant difference between patients and controls on BDI scores (*p* = 0.001), but not on BAI (*p* = 0.489) or PSS scores (*p* = 0.565). *Post hoc* t-tests, using a Bonferroni-corrected alpha level of 0.05/2 = 0.025 (each power was calculated using *α* = 0.025), revealed that patients’ BDI scores differed significantly from those of controls at baseline (t = 5.057, df = 38, *p* < 0.0005, Cohen’s *d* = 1.630, Power = 0.997), but not at the end of the study (t = 1.129, df = 38, *p* = 0.266, Cohen’s *d* = 0.364, Power = 0.129).

**Figure 1:**
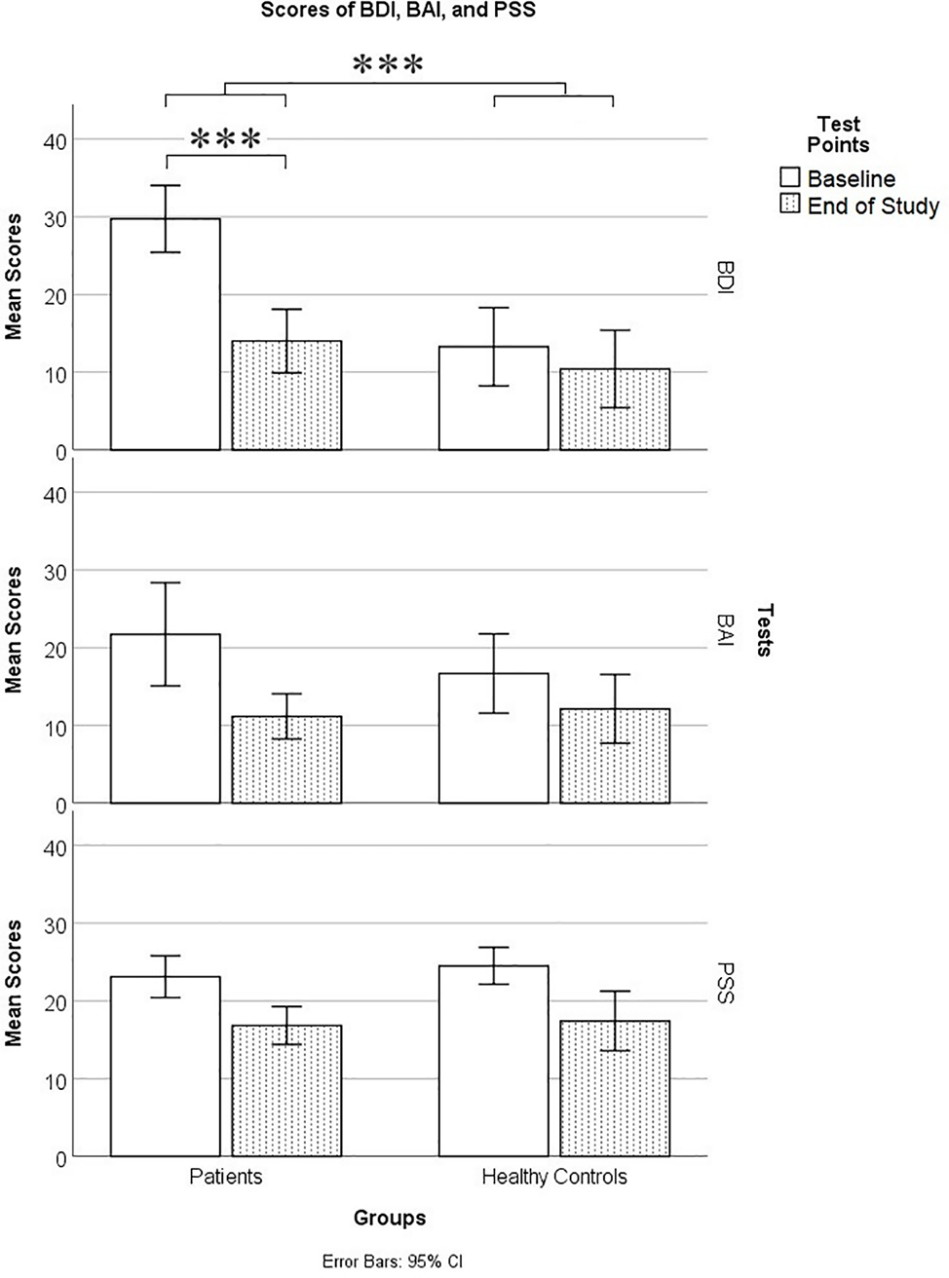
Mood scores on the Beck Depression Inventory II (BDI), Beck Anxiety Inventory (BAI), and Cohen’s Perceived Stress Scale (PSS) in patients and controls at baseline and end of study. *** means p ≤ 0.001.

A total BDI-II score of 29–63 is considered to be in the severely depressed range, 20–28 is moderate, 14–19 is mild, and 0–13 is minimal. In the patient group, at baseline, there were 10 in the severe range, five moderate, and three mild. At end of study, there was one patient in the severe range, three moderate, four mild, and 10 minimal. In the control group, at baseline, there were four in the severe range, two moderate, two mild, and 14 minimal. At the end of the study, there were two controls in the severe range, two moderate, one mild, and 17 minimal. As can be seen in **Figure 1**, overall, patients were significantly depressed at baseline but had recovered from depression by the end of the study.

### Cognitive Tests

**Figure 2:**
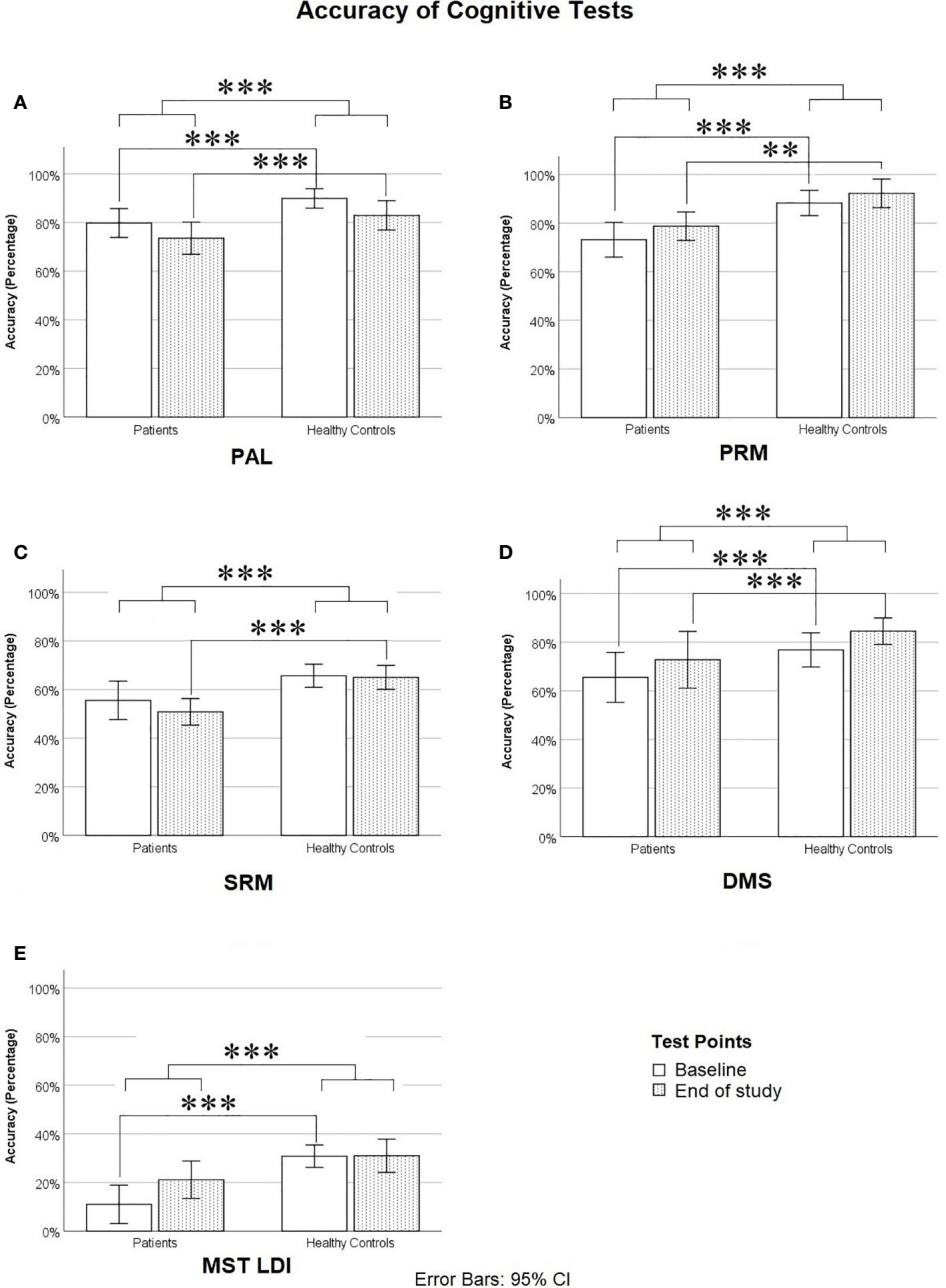
Accuracy on cognitive tests: **(A)** Paired Associates Learning (PAL, Average of PAL-6 and PAL-8), **(B)** Pattern Recognition Memory (PRM), **(C)** Spatial Recognition Memory (SRM), **(D)** Delayed Match to Sample task (DMS, Level 4), and **(E)** Mnemonic Similarity Test Lure Discrimination Index (the proportion of lures correctly called “Similar” minus the proportion of novel foils incorrectly called “Similar”). *** means p ≤ 0.001.

On cognitive scores, a repeated measures (group × time) MANOVA revealed significant main effects of group [*F* (6, 33) = 6.778, *p* < 0.0005; Wilk’s Λ = 0.448, Observed Power = 0.997], and time, *F* (6, 33) = 6.607, *p* < 0.0005; Wilk’s Λ = 0.454, Observed Power = 0.997, but no significant group by time interaction, *F* (6, 33) = 1.292, *p* = 0.288; Wilk’s Λ = 0.810, Observed Power = 0.433. Pairwise Bonferroni-corrected comparisons of marginal means within the MANOVA analysis showed that healthy controls were significantly more accurate than patients on PAL (*p* < 0.0005), PRM (*p* = 0.001), SRM (*p* < 0.0005), DMS (*p* < 0.0005), and MST (*p* = 0.001) (see **Table 3** and **Figure 2**), but patients did not differ from controls on the RVIP (*p* = 0.538). Therefore, RVIP was omitted from further analyses.

**Table 3:** Mood Scores (averages and standard deviations) and Cognitive Test Accuracy Scores (% correct and standard deviations).

Tests	Levels	18 Patients		22 Healthy Control Participants	
		Pre-Test	Post-Test	Pre-Test	Post-Test
Beck Depression Inventory II (BDI)		29.72 (8.65)	14.00 (8.23)	13.27 (11.36)	10.41 (11.24)
Beck Anxiety Inventory (BAI)		21.72 (13.35)	11.17 (5.84)	16.68 (11.51)	12.14 (9.98)
Cohen’s Perceived Stress Scale (PSS)		23.11 (5.41)	16.83 (4.90)	24.50 (5.35)	17.41 (8.64)
Rapid Visual Information Processing (RVIP)		74.44% (16.88%)	75.56% (29.55%)	68.64% (29.00%)	72.27% (32.50%)
Pattern Recognition Memory (PRM)		73.15% (14.38%)	78.70% (11.78%)	88.26% (11.76%)	92.23% (13.26%)
Spatial Recognition Memory Task (SRM)		55.56% (15.89%)	50.83% (11.02%)	65.68% (10.72%)	65.00% (11.13%)
Delayed Match to Sample (DMS)	1	78.89% (15.30%)	91.67% (9.85%)	94.55% (11.84%)	97.27% (5.51%)
	2	61.11% (16.41%)	62.22% (18.33%)	80.91% (13.77%)	87.73% (15.72%)
	3	59.44% (23.38%)	61.67% (25.03%)	73.64% (15.90%)	77.73% (17.16%)
	4	65.56% (20.64%)	72.78% (23.47%)	76.82% (15.85%)	84.55% (12.24%)
Paired Associate Learning (PAL)	1 (1)	100% (0)	100% (0)	100% (0)	100% (0)
	2 (2)	87.96% (17.90%)	94.44% (14.00%)	98.48% (4.90%)	95.08% (8.40%)
	3 (3)	91.05% (12.52%)	92.44% (11.66%)	94.14% (11.59%)	96.46% (7.18%)
	4 (6)	69.94% (10.41%)	77.93% (19.63%)	91.54% (14.71%)	91.31% (13.13%)
	5 (8)	73.56% (13.31%)	81.60% (9.21%)	82.94% (13.64%)	88.40% (11.39%)
Mnemonic Similarity Test (MST)	1 (New)	87.92% (15.19%)	93.70% (13.20%)	95.23% (5.76%)	93.37% (13.38%)
	2 (Old)	64.90% (18.22%)	64.14% (11.89%)	66.53% (10.69%)	63.84% (10.23%)
	3 (Similar)	19.07% (14.25%)	24.24% (15.08%)	35.12% (9.79%)	35.43% (12.96%)
	Lure Discrimination Index (LDI)	11.01% (15.88%)	21.14% (15.48%)	30.81% (10.39%)	31.00% (15.43%)

*Post hoc* t-tests, using a Bonferroni-corrected alpha level of 0.05/5 = 0.01 (each power was calculated using *α* = 0.01), showed that at baseline, healthy controls significantly outperformed patients on PAL (t = −4.356, df = 38, *p* < 0.0005, Cohen’s *d* = 1.411, Power = 0.952), PRM (t = −3.658, df = 38, *p* = 0.001, Cohen’s *d* = 1.150, Power = 0.811), DMS (t = −4.303, df = 38, *p* < 0.0005, Cohen’s *d* = 1.344, Power = 0.929), MST LDI (t = −4.743, df = 38, *p* < 0.0005, Cohen’s *d* = 1.478, Power = 0.969), and marginally on SRM (t = −2.398, df = 38, *p* = 0.028, Cohen’s *d* = 0.746, Power = 0.370). At the end of the study, healthy controls significantly outperformed patients on PAL (t = −3.048, df = 38, *p* = 0.004, Cohen’s *d* = 0.954, Power = 0.616), PRM (t = −3.375, df = 38, *p* = 0.002, Cohen’s *d* = 1.079, Power = 0.749), DMS (t = −4.035, df = 38, *p* < 0.0005, Cohen’s *d* = 1.253, Power = 0.883), and SRM (t = −4.024, df = 38, *p* < 0.0005, Cohen’s *d* = 1.280, Power = 0.899), but patients did not differ significantly from controls on the MST LDI, although there was a trend toward controls outperforming patients at the second time point (t = −2.008, df = 38, *p* = 0.052, Cohen’s *d* = 0.638, Power = 0.256). We also repeated the above MANOVA and *post hoc* comparisons of cognitive scores after removing the four control participants whose BDI scores fell into the severe range at either baseline, end of study, or both. The results followed the same overall pattern, including a significant difference between patients and controls at baseline but not at end of study on the MST, with one exception: patient-control differences on the DMS were no longer significant at either time point, although patients trended toward poorer DMS performance at baseline (*p* = .076).

As can be seen in **Figure 2**, at baseline, patients were impaired relative to controls on nearly all cognitive measures. On PAL, this impairment was particularly evident at the highest difficulty levels (PAL-6 and PAL-8), as can be seen in **Table 3**. Across the two time points, there was very little if any improvement in cognitive scores on all measures with the exception of the MST (see **Table 3** and **Figure 2**), where patients showed a considerable improvement from baseline to end of study and were only slightly but not significantly impaired relative to controls by the end of study on the Lure Discrimination Index (Bias-corrected performance on “Similar” lures).

Practice effects are a potential explanation of the improvement in MST scores from baseline to end of study. Alternatively, part or all of the improvement in MST scores could be due to a recovery of hippocampal function associated with recovery from depression. In an attempt to tease apart these two explanations, we divided patients into two groups, those who had a clinically significant improvement and those who did not, based on the standard clinical cut-off scores for the BDI-II. We defined a clinically significant improvement as an improvement of two or more levels, *i.e.*, in the severe range at baseline and minimal or mild range at end of study, or moderate at baseline and minimal at end of study. The MST LDI scores for the two groups are summarized in **Table 4**. If improvement in MST scores from baseline to end of study was merely due to practice effects, we would expect to see similar improvements in MST scores in the two groups.

**Table 4:** MST difference scores for patients in Low and High improvement groups.

BDI Improvement groups	N	Mean	95% Confidence Interval
Low Improvement	9	6.47%	−0.502–17.96%
(Patients improved 0 or 1 levels on BDI scores at the end of study compared to baseline)
High Improvement	9	13.79%	4.73–22.85%
(Patients improved 2 levels on BDI scores at the end of study compared to baseline)

**Figure 3:**
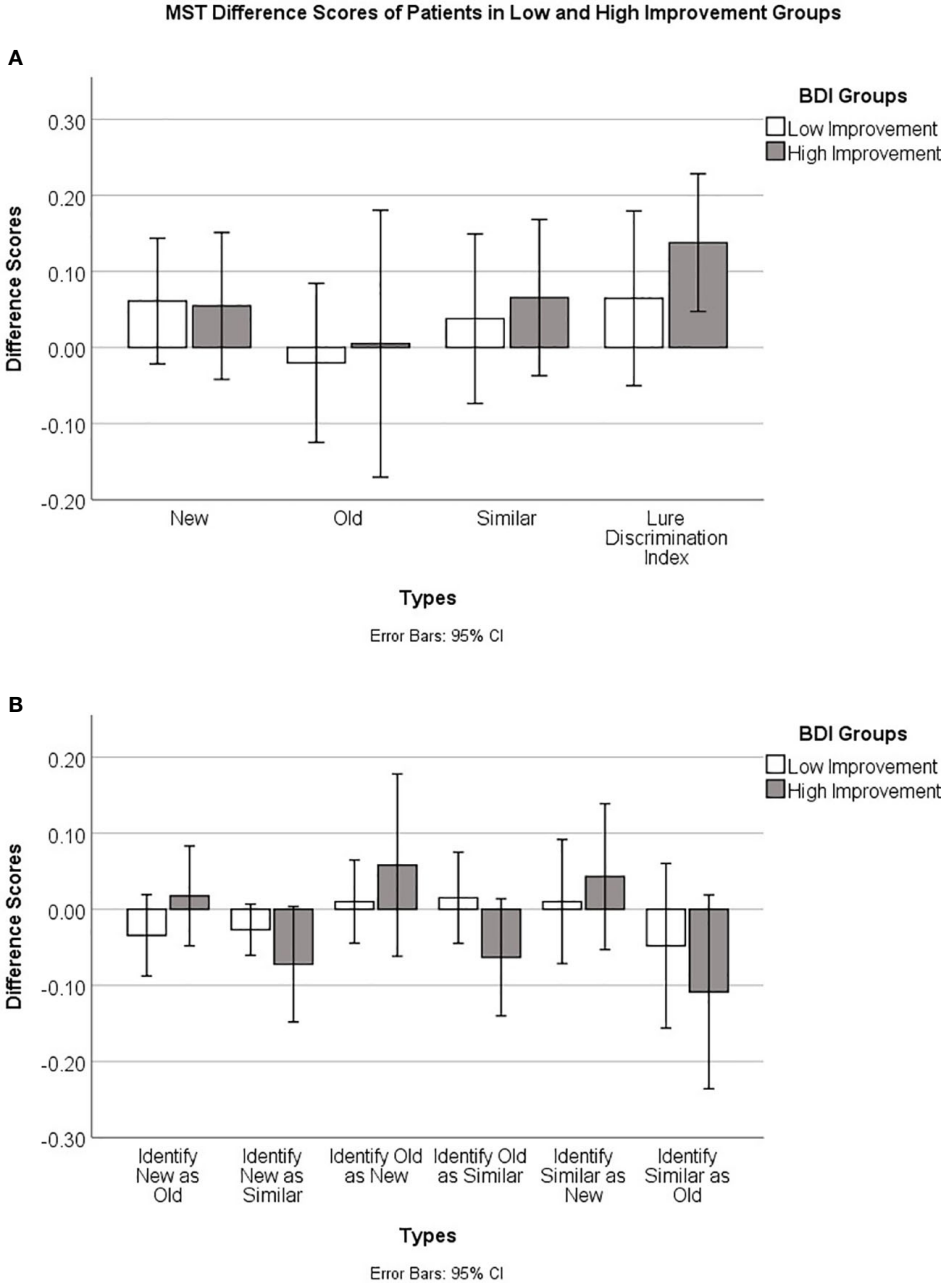
**(A)** Baseline to end of study accuracy difference scores (End of study–Baseline), and 95% confidence intervals, on the Mnemonic Similarity Test for patients who had lower *versus* higher clinical improvement based on BDI-II clinical cut-off scores (see text for details). **(B)** Baseline to end of study error difference scores (End of study–Baseline) and 95% confidence intervals for the different error types on the Mnemonic Similarity Test for patients who had lower *versus* higher clinical improvement.

We tested the null hypothesis that in each of the two groups, the change in MST LDI scores was not significantly greater than zero. Independent one-sample t-tests, using Bonferroni-corrected alpha levels of 0.05/2 = 0.025, revealed that patients who had clinically significant improvement showed an improvement on MST LDI scores that was significantly greater than zero (t = 3.510, df = 8, *p* = 0.008), while those with lower clinical improvement did not (t = 1.299, df = 8, *p* = 0.230). This can be seen in **Figure 3A**), which shows the difference scores in MST accuracy. A positive difference score indicates an improvement from baseline to end of study. For the group that showed higher clinical improvement, the 95% confidence interval is well above zero. In contrast, for the group that had less clinical improvement, the confidence interval spans the x-axis, indicating that some patients in this group improved on the MST while others did not. **Figure 3B** shows the proportions of each type of error made by the patients in the two groups, displayed as baseline to end of study difference scores. Here, a negative difference score means an improvement, as fewer errors were made at the end of the study relative to baseline. Patients who showed higher clinical improvement also tended to make fewer old-similar confusions as well as fewer mis-identifications of new items as similar at the end of the study time point relative to their baseline. The 95% confidence intervals for these error types cross the X-axis but just barely. These trends should be investigated further in a larger sample.

## Discussion

In general, our results are in line with previous research showing that people who suffer from depression have cognitive impairments; importantly, many of these deficits persist even after patients are in remission. Findings on cognitive deficits in remitted patients who had major depression are highly variable in the literature, as revealed in meta-analyses (10, 11). While in our study remitted patients showed lingering deficits on several memory tests, including the CANTAB-like Pattern Recognition Memory, Spatial Recognition Memory, Delayed Matching to Sample and Paired Associates Learning tasks, not all studies have reported memory deficits in major depressive remitters on these tasks. As noted previously, a major source of this variability is the number of previous depressive episodes (10), which is in turn correlated with the degree of hippocampal volume loss (15, 16). Some of the patients in the current study, although hospitalized for the first time, could have experienced prior episodes of depression, which would explain the broad memory deficits upon remission. Unexpectedly, we did not find any difference between healthy control participants and patients in the CANTAB-like Rapid Visual Information Processing test at either time point, whereas a meta-analysis by Rock et al. (11) reported large RVIP deficits in remitted patients. Additionally, while we did not observe a reaction time difference between patients and controls on the motor screening task (MOT), a processing speed impairment has been reported in unremitted (but not remitted) depressed patients, and this factor was a significant moderator of their memory impairments (35). In our study, the MOT may have been insufficiently sensitive to detect a processing speed deficit in patients, as their MOT accuracy scores were near ceiling.

Another potential explanation of our findings of broad memory deficits in remitted patients could be their attentional biases. Patients with remitted depression show attentional and memory biases toward negative information at the expense of positive information (12). Indeed, meta-analyses reveal that remitted patients show lingering deficits on some measures of memory, but are superior to healthy controls on identifying emotional facial expressions and remembering self-referring negative words (10). In our study, only neutral or positive stimuli were used, and remitted patients may have been less successful at encoding these items due to their information processing bias. Future studies could examine performance on versions of the memory tests used here, but incorporating a range of negative, neutral and positive stimuli.

Importantly, remitted patients in the current study showed recovery of function on the Mnemonic Similarity Task (MST) lure discrimination index, a test of high interference memory, such that their scores were much closer to, and not significantly different from, those of healthy controls at end of study. This finding is consistent with our prediction based on the neurogenic theory of depression, which posits that recovery from depression is dependent upon an increase in hippocampal neurogenesis. While neurogenesis cannot be assessed non-invasively in the living human brain, a large body of evidence from non-human animal models and indirect evidence from humans points to a critical role for neurogenesis in mediating high interference memory. For example, when neurogenesis is knocked down in rodents, *via* stress, binge alcohol exposure or irradiation, performance on a wide range of high interference memory tasks is disrupted [for a review, see Becker (27)]. Similarly, in otherwise healthy young adults, stress and depression scores and binge drinking levels are associated negatively with performance on high interference memory tasks including the MST and the Concentration Memory Task (28–30). Conversely, exercise up-regulates neurogenesis and improves performance on high interference tasks in rodents [for a review, see Becker (27)], and several weeks of aerobic exercise improves performance on the MST in humans (28, 36). Moreover, evidence from both humans and non-human animals points to a critical role for neurogenesis in mediating recovery from depression. In rodent models, anti-depressants reverse stress-induced neurogenesis knockdown, and blocking this recovery of neurogenesis also blocks the behavioural response to antidepressants (23, 37). Similarly, *post-mortem* studies of those with major depression reveal that patients who received anti-depressant treatment had increased neurogenesis and vasculature in the dentate gyrus of the hippocampus relative to the brains of untreated patients (38). We therefore predicted an improvement in performance on the MST in remitted patients. As predicted, we found that patients were impaired relative to controls at baseline, but not at end of study, on this high interference memory task, putatively correlated with neurogenesis levels. This finding is consistent with predictions of the neurogenic hypothesis of depression, although it does not rule out the possibility that other mechanisms may have contributed to patients’ recovery.

It is somewhat paradoxical that remitted patients were unimpaired on a putatively hippocampal neurogenesis-dependent memory test, the MST, while being impaired on a variety of other memory tests that seem to implicate hippocampal volume loss. A potential explanation is that their recovery from depression was associated with a recovery of neurogenesis specifically within the dentate gyrus of the hippocampus, in spite of loss of volume in other hippocampal sub-regions that also support memory. Neuroimaging studies in healthy individuals performing the MST reveal that viewing “high interference items”—images that are highly similar to previously studied items—generated a novelty response in the DG/CA3 region of the hippocampus (these sub-regions could not be resolved separately in this study) but not other hippocampal sub-regions (39). This is consistent with evidence from rodents that subtle changes to an environment evoke distinct patterns of activation in the DG, while larger environmental changes were detected and represented in the CA3 (40). The implication is that the DG is important for memory of highly specific information and differentiating new items from previous highly similar memories, whereas other hippocampal regions are important for more general or less specific aspects of recognition and recall. Moreover, as noted above, anti-depressant treatment is associated with an increase in dentate gyrus volume, vasculature and neurogenesis in the hippocampi of depressed patients upon autopsy (38). Thus, it is plausible that remitted patients had restoration of neurogenesis in the dentate gyrus associated with improved performance on the MST. On the other hand, they may have had structural changes in other hippocampal sub-regions that did not resolve upon remission, consistent with evidence of cumulative hippocampal volume loss in MDD patients in association with total lifetime illness duration (15, 16). More specifically, a high-resolution 7T MRI study in patients with MDD revealed hippocampal volume loss in hippocampal sub-regions CA3 and CA1 that correlated with symptom severity (41). Establishing whether these region-specific volume losses show recovery upon remission requires high resolution longitudinal studies in depressed patients, from acute onset through to remission.

It is worth noting that in our control sample of young adults, participants varied in their depression (BDI-II) scores, with the majority falling into the minimal range, but several falling into each category including four in the severe range (four at baseline and two at the end of the study). Based on our past research, this is typical in samples of young adults. For example, in a sample of 155 “healthy” university students who had never been diagnosed with depression, nine scored in the severe range on the BDI-II (42). Interestingly, those in the severe range, relative to the remaining participants, were impaired on the DMS but not on other CANTAB tests. The DMS was suggested to be putatively sensitive to neurogenesis, as the delay creates a high degree of interference. Broadly consistent with these findings, in the present study, when control participants in the severe range on the BDI were excluded from our analyses, the overall pattern of results did not change, with the exception of the delayed match to sample (DMS), where patients were marginally impaired relative to controls at baseline but not at the end of the study. This pilot study has a number of limitations. First and foremost, the sample size is relatively small, increasing the risk of overestimating effect sizes (type I errors). Second, we did not have access to the individual patient charts, which would have included important clinical history variables such as previous depressive episodes and the specific treatments that patients were receiving in hospital; these could be possible confounding factors. Third, no long-term follow-up assessment was conducted after patients had been discharged. Future studies should track a larger group of patients longitudinally throughout their hospitalization period as well as post-discharge.

Another potential limitation of our study is that there may have been practice effects from baseline to the end of the study testing. There are well documented practice effects on many of the CANTAB tests [see *e.g.* Karlsen et al. (43)]. However, practice effects, if anything, should work against our ability to detect impairments in remitted patients’ cognitive performance. Thus, the observation that patients were still significantly impaired on nearly all CANTAB tests at the end of study time point cannot be explained by practice effects. In contrast, relative to controls, patients were not significantly impaired at end of study on the MST lure discrimination index (bias-corrected performance on similar items). In this case, practice effects could be an explanation for the lack of a significant impairment on the MST high interference items in remitted patients relative to controls. Moreover, we used the same images in the MST at both baseline and end of study. This might have made the repeated or old items easier to discriminate as they would be seen for the second time in the end of study test. On the other hand, having previously seen the old, similar and new items at baseline could increase the familiarity of all three stimulus types, thus making the three stimulus types more difficult to differentiate at the end of study. This should be particularly true of the novel foils, where pre-exposure to the MST foils at baseline should have made them much more difficult to discriminate as being “New”, because they would have been somewhat familiar during the second test. Importantly, however, the sub-group of patients who showed a clinically significant improvement (defined here as improving by two levels on their BDI scores, either moving from the severe to the mild or minimal range, or from the moderate range to the minimal range) improved significantly on the MST LDI, while patients who did not show a clinically significant improvement (they improved by 0–1 levels) did not show a baseline to end of study improvement on the LDI. This strongly suggests that the improvement in MST performance in patients was directly related to their changes in mood, and not merely to practice effects. Nonetheless, future studies should be run with a larger sample size, using distinct sets of MST images in the pre- and post-tests, to further tease apart the possible explanations for our findings.

Many factors that were not assessed in the present study could shed further light on the mechanisms associated with recovery from MDD. For example, high-resolution MRI could permit assessment of regional hippocampal volumetric changes in relation to cognitive and mood changes. The prediction is that improvement in MST performance in remitted patients would be associated with recovery of hippocampal volume in the dentate gyrus, while lingering memory deficits would be associated with less or no recovery of volume loss in CA3 and CA1 regions. Plasticity markers including BDNF and VEGF could be assessed as well; in rodent hippocampi, these biomarkers are found to regulate levels of neurogenesis (44, 45). BDNF, in particular, has been found to mediate the exercise-induced increase in neurogenesis [for a review, see Liu and Nusslock (46)] and to co-modulate both neurogenesis and depressive behaviors in rodents (47), while the anti-depressant effects of BNDF require VEGF signalling (48). It is predicted that BDNF and VEGF levels should increase together with neurogenesis, particularly in the patients who showed the greatest degree of clinical improvement. Other aspects of cognitive function that are known to be altered in remitted MDD patients and were not investigated in the current study include emotional processing biases and autobiographical memory specificity (12). Finally, we did not have access to detailed records on specific pharmacological and psychotherapeutic treatments that patients received during hospitalization. Various treatments could differentially impact neurogenesis-specific functions associated with recovery from depression.

In summary, we found evidence of lingering long-term memory deficits in remitted patients across multiple tests of memory, broadly consistent with findings from neuroimaging studies in humans with major depressive disorder showing reduced hippocampal volume. Importantly, however, remitted patients, but not healthy controls, showed improved scores on the Mnemonic Similarity Task, a high interference memory test that is a putative correlate of neurogenesis. This novel finding is consistent with the neurogenic theory of depression and findings from studies of animal models showing that recovery of neurogenesis is linked to recovery from depression.

## Data Availability Statement

The datasets generated for this study are available on request to the corresponding author.

## Ethics Statement

All participants voluntarily chose to join the study, provided written consent, and were free to withdraw at any time. The study was reviewed and approved by the ethics committees in the Changchun Mental Hospital and Northeast Normal University (2017002).

## Author Contributions

XH: conceptualization, data curation, formal analysis, funding acquisition, methodology, resources, software, supervision, validation, visualization, writing original draft and review and editing. YW: investigation, project administration. YZ: resources, investigation.

SB: funding acquisition, resources, software, conceptualization, writing—review and editing.

## Conflict of Interest

The authors declare that the research was conducted in the absence of any commercial or financial relationships that could be construed as a potential conflict of interest.
